# Assessing Sustainability in the Textile Sector: A Review of LCA, LCC, and S-LCA Methodologies with a Focus on Polymeric Textile Materials and Circular Strategies Along with Future Perspectives

**DOI:** 10.3390/polym18040534

**Published:** 2026-02-21

**Authors:** Anastasia Anceschi, Raffaella Mossotti, Alessia Patrucco

**Affiliations:** Institute of Intelligent Industrial Technologies and Systems for Advanced Manufacturing (STIIMA), Italian National Research Council (CNR), Corso G. Pella 16, 13900 Biella, Italy; raffaella.mossotti@cnr.it (R.M.); alessia.patrucco@cnr.it (A.P.)

**Keywords:** sustainability assessment, Life Cycle Assessment (LCA), Life Cycle Costing (LCC), Social Life Cycle Assessment (S-LCA), textile supply chain, circular economy, polymeric textile fibers

## Abstract

The textile industry is facing increasing pressure to improve its sustainability performance across environmental, economic, and social dimensions. A substantial share of textile production relies on polymer-based fibers, such as polyester, polyamide, and acrylics, whose production, use, and end-of-life management raise significant sustainability challenges. In this context, life cycle-based assessment tools have become essential for supporting informed decision-making and guiding the transition toward more circular textile systems. This review critically examines the application of Life Cycle Assessment (LCA), Life Cycle Costing (LCC), and Social Life Cycle Assessment (S-LCA) within the textile sector, with a specific focus on polymeric textile materials and circular economy strategies. The analysis highlights the strengths and limitations of each methodology, emphasizing persistent challenges related to system boundary definition, data availability and quality, methodological heterogeneity, and limited comparability across studies. Particular attention is given to how methodological choices influence the robustness and interpretability of sustainability outcomes, especially when assessing circular solutions for polymer-based textiles. The review reveals that, despite their conceptual complementarity, LCA, LCC, and S-LCA are often applied in a fragmented manner, limiting their integration into holistic sustainability assessments. Overall, this work underscores the need for greater methodological alignment and integrated frameworks to enhance the decision-making relevance of life cycle-based tools and to effectively support sustainable and circular transitions in the textile industry.

## 1. Introduction

The textile sector is a major driver of the global economy, with an estimated market value of €848.78 billion. Apparel dominates the industry, representing about 75% of the market, followed by technical textiles (12%) and household goods (9%) [1]. A significant share of the textile sector relies on synthetic polymer-based fibers such as polyester, polyamide, acrylic, and elastane, which currently dominate global fiber production. Indeed, the current system is predominantly driven by ultra-fast fashion production that relies heavily on non-renewable resources, primarily derived from fossil fuels [2]. This model has led to a significant increase in demand for synthetic fibers, which has further contributed to resource depletion and environmental concerns. Beyond the concerns related to raw material use, textile manufacturing processes such as dyeing and finishing have emerged as significant contributors to environmental concerns, particularly in terms of wastewater contamination [3]. Furthermore, the energy demands of the textile industry, particularly for processes such as washing, dyeing, and drying, are frequently supported by fossil fuels. This is particularly evident in major textile-producing countries, which continue to place significant reliance on coal for the energy needs of their manufacturing operations [4,5]. The issue of end-of-life textile waste has been recognized as a major factor contributing to environmental challenges. The predominant methods of disposal, landfilling and incineration, account for the majority of textile waste management, with less than 1% of discarded textiles currently being recycled into new textile products [6].

**Figure 1:**
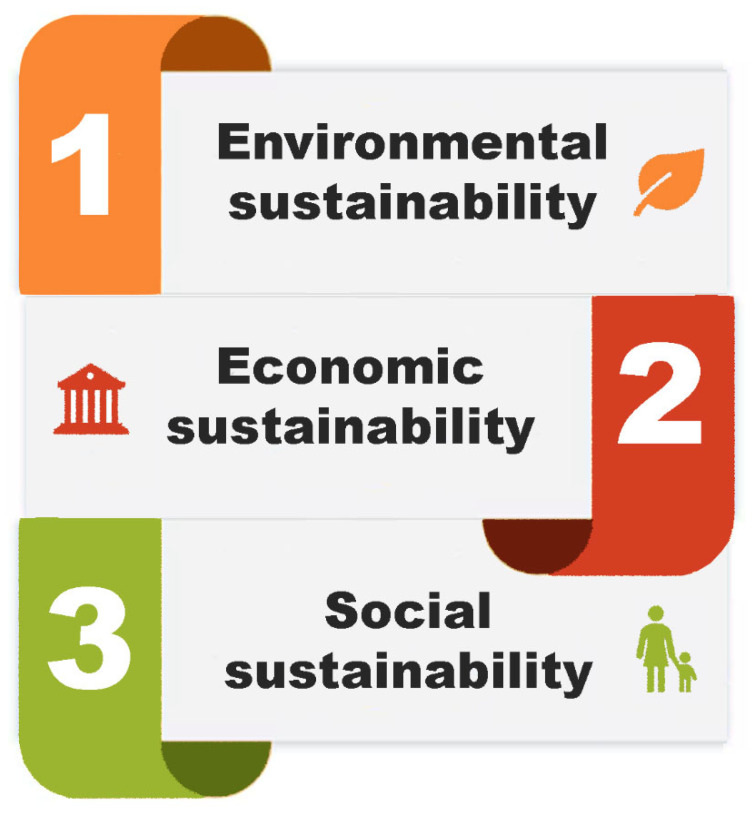
Core dimensions of sustainability.

Within this context, sustainability in the textile industry is a multifaceted and evolving concept, encompassing environmental protection, economic viability, and social responsibility. It is commonly framed around three interrelated pillars: environmental, economic, and social sustainability, as shown in Figure 1.

Environmental sustainability focuses on preserving ecosystem integrity, maintaining ecological functionality, and minimizing the overexploitation of natural resources, particularly in resource- and energy-intensive production processes [7]. Economic sustainability refers to production systems capable of meeting current market demands while maintaining long-term viability without depleting natural or financial capital [8]. Social sustainability, in turn, emphasizes fair labor practices, safe working conditions, equitable access to opportunities, and respect for human rights throughout the value chain.

**Figure 2:**
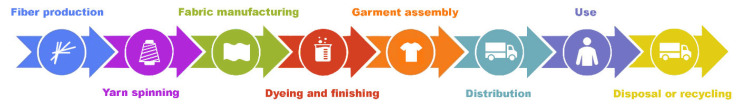
Textile supply chain.

In the textile sector, these sustainability dimensions are tightly interconnected and require a comprehensive transformation of the complex and globalized value chain [9]. A textile process, typically, starts with the production of fibers, which can be natural, such as cotton or wool; artificial, such as viscose or lyocell; or synthetic, such as polyester or polyamide. This is followed by yarn spinning, fabric manufacturing through weaving or knitting, dyeing and finishing, and subsequently, garment tailoring. The final stages include distribution, use, and eventual disposal or recycling, as illustrated in Figure 2.

The textile value chain is a key area for sustainability assessment and improvement [10]. It refers to the adoption of innovative technologies, the use of environmentally friendly materials, and more responsible practices aimed at reducing ecological burdens [11]. This involves shifting towards renewable and sustainable raw materials, optimizing water and energy use, and adopting cleaner, low-impact production processes and ensuring ethical approaches that respect human rights and well-being in the workplace. In this context, the textile industry faces significant challenges, but it also has opportunities to transform its practices and contribute to a more circular and resilient global economy [12]. Over the past two decades, growing attention has been directed toward textiles and apparel produced with reduced environmental and social impacts. This shift has been driven by a wide range of actors, including sustainability-aware consumers, major international brands and retailers, non-governmental organizations and advocacy initiatives, as well as regulatory bodies and public institutions, such as the EU REACH regulation [13]. Given the complex, multidimensional nature of sustainability in the textile sector, robust and structured analytical tools are required to support informed decision-making. Among the most widely applied approaches are Life Cycle Assessment (LCA), Life Cycle Costing (LCC), and Social Life Cycle Assessment (S-LCA), which, respectively, address the environmental, economic, and social dimensions of sustainability [14,15]. These methodologies are grounded in life cycle thinking and aim to capture impacts across the entire textile value chain, from raw material extraction to end-of-life management of production [16,17].

However, despite their increasing adoption, their application within the textile sector remains heterogeneous, often characterized by inconsistent system boundaries, data limitations, and limited comparability across studies, thereby constraining their effectiveness in supporting integrated sustainability assessments [14,15]. Accordingly, this review is conceived as a narrative and critical review with the objective of critically analyzing how life cycle-based sustainability assessment tools such as LCA, LCC, and S-LCA are applied within the textile sector, with particular attention to polymer-based materials and circular economy strategies. Moreover, this work focuses explicitly on polymer-based textiles within circular economy pathways and aims to evaluate how methodological choices affect the robustness, comparability, and decision-making relevance of sustainability assessments.

To structure the discussion, the review is guided by the following questions:

(1) What are the conceptual foundations and methodological principles of LCA, LCC, and S-LCA in the context of sustainability assessment?
(2) How are LCA, LCC, and S-LCA currently applied in the textile sector?
(3) limitations and research gaps hinder the effective use of life cycle-based tools in supporting circular strategies for polymer-based textiles?
(4) To what extent do existing studies allow for comparability and integration across environmental, economic, and social dimensions?

To address these questions, this review adopts a critical narrative approach. The literature was analyzed and interpreted with the aim of identifying recurring methodological patterns, assumptions, and limitations in the application of LCA, LCC, and S-LCA within the textile sector. Furthermore, the literature considered in this review was identified through targeted searches in major scientific databases, including Scopus, Web of Science, and Google Scholar. Indicative keywords included combinations of life cycle assessment, life cycle costing, social life cycle assessment, textile sector, polymeric fibers, and circular economy. The selection of studies was guided by their relevance to life cycle-based sustainability assessment in textiles, with particular attention to methodological approaches, system boundary definitions and applications to polymer-based materials and circular strategies.

## 2. What Are the Conceptual Foundations and Methodological Principles of LCA, LCC, and S-LCA in the Context of Sustainability Assessment?

Life cycle-based sustainability assessment tools are rooted in life cycle thinking, which aims to capture environmental, economic, and social impacts across the entire value chain. LCA, LCC, and S-LCA share common methodological principle, such as system boundary definition, functional units, and life cycle-wide data analysis, while addressing distinct sustainability dimensions, thereby enabling a holistic assessment of product and process performance [17,18]. The following sections therefore examine each methodology separately, highlighting both their potential and their limitations in textile sustainability assessment.

### 2.1. Life Cycle Assessment

LCA is a scientific methodology used to identify, quantify, and evaluate the environmental impacts associated with a product, process or service in all stages of its life. Often described as a “cradle-to-grave” approach, LCA may consider every phase of a product’s life, from the extraction of raw materials (the “cradle”) to manufacturing, distribution, use, and final disposal (the “grave”) [19]. In addition, a ‘cradle-to-cradle’ life cycle analysis may also consider the recycling stage, providing information on whether or not the product can be recycled and, if so, how and with what impact [20]. LCA can also be applied to individual processes or systems, and it exists in several variants depending on the scope of the analysis [21,22]. LCA is particularly relevant in the context of textiles due to the complex and environmentally impactful nature of production processes. Indeed, it enables stakeholders to identify environmental critical points and prioritize interventions [23].

**Figure 3:**
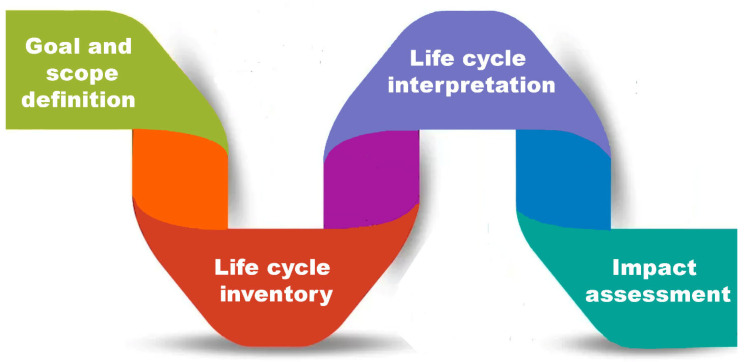
LCA stages.

The methodology for LCA is formalized through a series of ISO standards, with ISO 14040 (ISO 14040:2006 Environmental management-Life cycle assessment—Principles and framework, ISO, International Standard, 2006) and ISO 14044 (ISO 14044:2006 Environmental management-Life cycle assessment—Requirements and guidelines, ISO, International Standard, 2006) being the most widely recognized and accepted. These standards establish the foundation for conducting a robust LCA study, including its principles, framework, and requirements. Typically, a complete LCA study consists of four main phases, as illustrated in Figure 3, which provide a systematic structure for analyzing the sustainability performance of a product or process.

There are also several variants based on the LCA methodology [24]. Depending on the scope and objectives of the study, a variety of approaches can be used to assess the environmental impact of a product or process. The most commonly used methods are the Intergovernmental Panel on Climate Change (IPCC) framework, ReCiPe, and the CML baseline [6]. Other methodologies, such as TRACI or Eco-Indicator, have also been used in specific cases. The most frequently considered impact category in LCA studies is Climate Change or Global Warming Potential (GWP).

While LCA is primarily used to evaluate environmental impacts, its conceptual framework is also adaptable to broader sustainability assessments. This has led some companies to develop customized internal LCA guidelines tailored to their specific sustainability strategies. Therefore, sector-specific guidelines have been introduced to ensure consistency and objectivity across industries [11]. One example is the Product Environmental Footprint (PEF) methodology, developed by the European Union [25]. The PEF method aims to provide a standardized approach to evaluating environmental impacts from the beginning to the end of a product’s life cycle.

### 2.2. Life Cycle Cost Analysis

In terms of economic sustainability, one of the most widely applied tools is LCC. This methodology evaluates the total cost of a product or service throughout its entire life cycle, including acquisition, operation, maintenance, and end-of-life disposal [26]. While conventional cost assessments focus only on initial expenses, the LCC provides a more comprehensive assessment by accounting for long-term operational and overhead costs, as well as potential economic benefits. Therefore, LCC can analyze the economic performance of materials, processes, and products over their lifetime. For instance, even though eco-friendly products or manufacturing techniques might seem more costly at first, LCC can demonstrate that these options frequently lead to decreased overall expenses because of enhanced energy efficiency, decreased waste or reduced disposal charges [27]. This perspective holds significant value within green public procurement, where decision-makers must evaluate not only short-term benefits but also the broader long-term economic impacts. Additionally, LCC serves a strategic function in advancing sustainable products. In some cases, it can demonstrate the financial viability of environmentally responsible products, often revealing them to be cost-effective or even more economical throughout their entire life cycle. This contributes to the mitigation of the conventional belief that green products invariably involve higher costs [28]. Consequently, it can function as a persuasive instrument, encouraging both public and private sector stakeholders to invest in more sustainable solutions, thereby supporting broader sustainability goals across the supply chain.

### 2.3. Social Life Cycle Analysis

**Figure 4:**
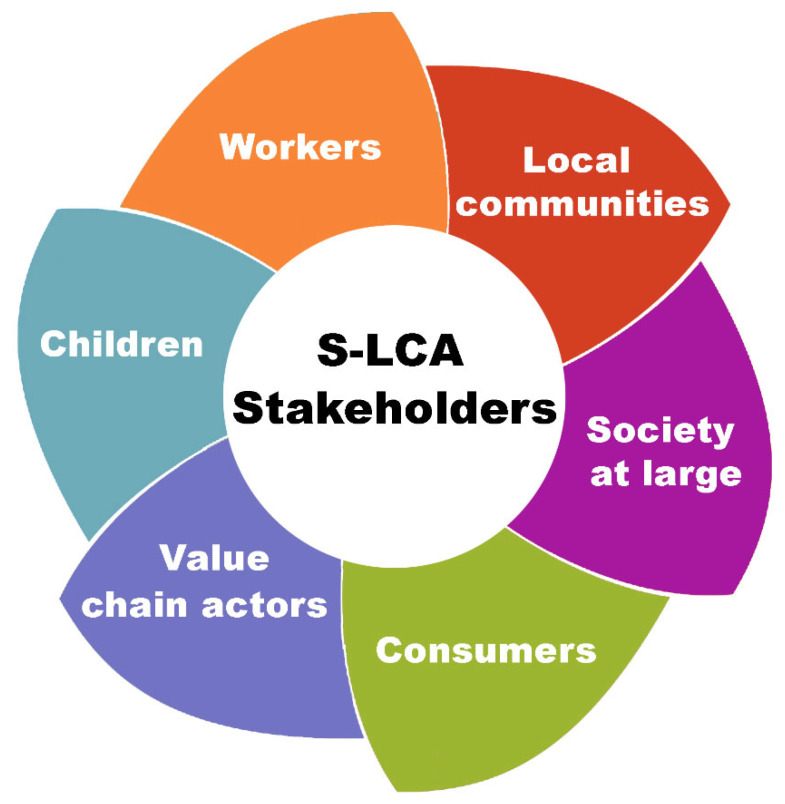
The six stakeholder categories typically considered in S-LCA.

As regards social sustainability, it can also be evaluated using a life cycle approach, most notably through Social-LCA [29]. This methodology extends the traditional LCA framework by incorporating social and socio-economic aspects into the analysis of products and processes. S-LCA is designed to identify and assess the potential positive and negative social impacts across all stages of a product, from raw material to end-of-life [30]. The S-LCA methodology is data-driven, incorporating a blend of generic and specific data. The indicators used in this framework may assume quantitative, semi-quantitative, or qualitative forms, contingent upon the scope and contextual framework [29,30]. It typically addresses social issues such as workers’ rights, occupational health and safety, community well-being, and fair-trade practices. S-LCA can be applied independently or in combination with LCA and/or LCC, enabling a more holistic assessment of the overall sustainability performance of product manufacturing. The textile industry, in particular, faces considerable challenges in relation to social sustainability [11]. A multitude of concerns have been identified, including toxic working conditions, inadequate salaries, child labor, and violations of labor rights. These issues are especially prominent in emerging economies, where a substantial share of textile manufacturing activities is concentrated [31]. Although the United Nations Environment Program (UNEP) and the Society of Environmental Toxicology and Chemistry (SETAC) have established guidelines for S-LCA, its practical implementation is still limited and continues to develop [32]. A literature review reveals the identification of two primary methodological frameworks for conducting the impact assessment phase in S-LCA: the “performance reference point” approach and the “impact pathway” approach. The first method involves evaluating stakeholder conditions at each phase of a product, process, or service to establish a benchmark for performance assessment. The impact pathway approach applies cause-and-effect models, similar to those in environmental LCA, to link activities with specific social impacts and outcomes [33]. These methodological frameworks include guidelines that identify six main stakeholder categories as reported in Figure 4.

Each stakeholder group represents a distinct perspective on social sustainability, reflecting the different roles and vulnerabilities present throughout the life cycle [32]. Within each of these stakeholder categories, a set of sub-categories is defined to enable a more detailed and targeted assessment of social impacts. These sub-categories correspond to specific social themes or issues, such as fair salary, welfare, community involvement, or education accessibility. Each sub-category is associated with one or more indicators, which provide measurable criteria useful for evaluating performance or identifying risk in relation to a specific process or product. These indicators enable the conversion of qualitative social concerns into measurable assessment outcomes, facilitating consistent and transparent decision-making within the S-LCA framework.

In conclusion, the LCA, LCC and S-LCA provide a robust and complementary framework for evaluating the environmental, economic, and social aspects of sustainability. When applied individually or in combination, these methodologies permit a holistic analysis of the impacts associated with textile production and consumption throughout the entire life cycle. Adopting them helps spot discrepancies, reduce drawbacks, and encourage sustainability across the value chain. Consequently, the following sections will delve more specifically into the LCA, LCC, and S-LCA within the textile industry, with a focus on key impact areas.

## 3. How Are LCA, LCC, and S-LCA Currently Applied Across Different Stages of the Textile Value Chain?

As sustainability becomes a central concern in the textile industry, it is imperative to possess a robust, reliable and science-based framework for assessing the sustainability of textile products. In particular, given that polyester and other synthetic polymer fibers represent a major portion of textile production, LCA plays a critical role in assessing their environmental impacts, particularly regarding fossil resource use and end-of-life scenarios.

Regarding the environmental impact of fiber production, silk and wool have been shown to contribute most significantly, primarily as a result of the scouring processes integral to their manufacturing. Among natural fibers, flax and jute have been demonstrated to exhibit the lowest carbon footprint, with the lowest CO_2_.equivalent emissions for fiber production, according to Fonseca et al. [6]. In the context of yarn production, the stage of spinning long fibers is typically identified as the most impactful [34]. Nevertheless, a lack of standardized and comparable data poses a significant challenge in comparing the environmental impacts of spinning across different fiber types.

More generally, there is a scarce availability of comprehensive datasets concerning yarn production in the existing literature on textile LCA. In an effort to address this gap, studies by Muthukumaran et al. and Periyasamy et al. compared the environmental impacts of final products made from polyester and cotton [35,36]. Surprisingly, both studies found that cotton-based products exhibited a higher environmental impact than polyester, generating 29.53 kg CO_2_ eq and 19.62 kg CO_2_ eq, respectively. These results are primarily attributable to the consideration of effects related to raw material procurement, fabric production, cutting, sewing, finishing processes, and logistics. It should be noted, however, that both studies omit consideration of the end-of-life phase for the products. This omission may substantially affect the assessment of overall sustainability, especially in the case of synthetic fibers such as polyester, which are known to persist in the environment following disposal. Indeed, when considering a cradle-to-gate approach, polyester emerges as one of the fibers with the highest environmental impacts. For example, the production of 1 kg of polyester, assuming it is used for at least one year, results in emissions of approximately 114.23 kg CO_2_ eq [37]. However, other studies report significantly lower values. For instance, Horn and colleagues found that the production of a polyester T-shirt has an impact of only 0.16 kg CO_2_ eq. Similarly, wool presents a wide range of reported environmental impacts. For example, Bech et al. estimated that the production of a wool T-shirt, assuming use over a six-month period and a closed-loop recycling in the end-of-life scenario, results in 91.72 g CO_2_ eq [38]. In contrast, Wiedemann et al. calculated that a wool sweater, with reuse considered at the end-of-life stage, generates 0.53 kg CO_2_ eq [39]. These discrepancies demonstrate the inherent variability in LCA results for textile products, which may stem from variations in system boundaries, assumptions about product lifespan, functional units, and end-of-life scenarios. This highlights the importance of establishing standardized and transparent methodologies to promote comparability and reliability across studies within the textile sector.

In light of the significant environmental impact associated with the textile sector, recent studies have begun to focus more closely on the role of textile waste within LCA frameworks. Indeed, researchers have identified the necessity of incorporating end-of-life stages into LCA studies to comprehensively assess the environmental impact of textile products. In response to this growing concern, European Union policies are now encouraging Member States to implement separate collection systems for textile waste, with the aim of preventing it from being landfilled or incinerated. Moreover, the EU is proactively fostering the development of sustainable hubs and innovative technologies to enhance the collection, sorting, and recycling of textile waste. In addition to technological solutions, public campaigns and policy initiatives are also promoting systems that support the repair and reuse of textile products. The objective of these efforts is twofold: first, to extend product lifespans, and second, to reduce the overall volume of waste, thereby promoting a more circular textile economy [40].

In the context of LCA studies on textile waste management, Dahlbo et al. and Farrant et al. investigated the potential environmental impacts of textile waste collection and sorting. The results of their studies indicate that the environmental impact of these activities is generally negligible, as they contribute to the reduction in the demand for virgin raw materials [41,42]. Dahlbo and colleagues examined various end-of-life scenarios, including recycling, reuse, and incineration with energy recovery, and found that reuse resulted in lower environmental impacts across all evaluated categories. This benefit is particularly pronounced when the reuse occurs within the same country where the textile was originally collected, thereby minimizing transportation emissions. These conclusions have been supported by other studies [43,44]. When reuse is not feasible, mechanical recycling, especially for natural fibers, emerges as the most environmentally favorable option, as highlighted by Moazzem and colleagues [45]. In addition to reuse and mechanical recycling, chemical recycling has also been explored as an end-of-life strategy for textile waste. Nonetheless, chemical recycling processes frequently exhibit a higher environmental impact, largely attributable to the intensive use of chemicals and energy inputs [40]. Among the various textile waste management options, the worst environmental performance is typically associated with thermal recycling to produce solid fuel, as demonstrated in the study by Semba et al. [46]. Lastly, incineration with energy recovery has also been evaluated as a potential strategy, though it generally results in higher emissions compared to other approaches [40]. As indicated in Table 1, various studies have explored the prospect of circular approaches in the context of textile end-of-life products.

**Table 1:** The effects of the circular options on the environmental impacts.

Functional Units	Circular Approach Used	Effect on Environmental Impacts	Reference
Lifecycle of a polyester workwear jacket for at least 4 years	PET from bottles	Climate Change: 18% reduction in greenhouse gas emissions Eutrophication: 23% reduction in nutrient release Ecotoxicity: 25% reduction in toxic substances Water Depletion: 38% reduction in freshwater consumption	[47]
Lifecycle of a mattress made of natural fiber for at least 10 years	Reuse of springs and frame Ethanol conversion Fiber recycling	Climate Change: 6–52% reduction in greenhouse gas emissions depending on the circular approach used.	[48]
Lifecycle of a polyester t-shirt for single use	Reuse Remanufacture Recycling	Climate Change: 8–33% reduction in greenhouse gas emissions Eutrophication: 5–41% reduction in nutrient release Water Depletion: 5–18% reduction in freshwater consumption	[49]
Lifecycle of cotton jeans for 200 uses	Extended use Reuse Recycling	Climate Change: 1–63% reduction in greenhouse gas emissions	[50]
Lifecycle of a roller-towel for single use	Reuse Recycling	Climate Change: 0–24% reduction in greenhouse gas emissions	[51]
Lifecycle of insulation panel for cars for at least 10 years	Manufacturing with recycled cotton	Climate Change: 42% reduction in greenhouse gas emissions Water Depletion: 23% reduction in freshwater consumption	[52]
Lifecycle of a polyester/wool sweater for single use	Extended use Manufacturing with recycled wool	Climate Change: 69–75% reduction in greenhouse gas emissions Water Depletion: 48–73% reduction in freshwater consumption	[53]

Among the various circular strategies, extending product life consistently provides significant environmental benefits. Research by Zamani et al. indicates that environmental impacts are reduced when garments are reused frequently, collected from local points, and transported with low-emission methods [54]. In addition, studies have demonstrated that reusable textiles generally have a lower environmental impact than disposable textiles, despite the significant energy and water consumption associated with laundry operations [55,56,57]. Reuse typically offers greater advantages than recycling, since recycling processes may necessitate the use of additional virgin materials, energy, and occasionally solvents or chemicals. However, the incorporation of strategies like component reuse in conjunction with fiber recycling can yield superior environmental results. For instance, the reuse of metal components and the recycling of textile fibers for production, such as mattress, can reduce environmental impacts by up to 90% [48].

Furthermore, Van der Velden and colleagues provide valuable insights into the environmental implications of textiles made by different materials, including cotton, PET, nylon, acrylic, and elastane [58]. The objective of their study is to identify which materials and life cycle stages contribute most significantly to environmental impact. Their analysis was based on data from existing literature, databases, and emission records from both the Dutch government and textile companies. Their study revealed that among the materials examined, acrylic and PET demonstrated the smallest environmental impacts, followed by elastane, nylon and cotton. Their study also found that spinning and weaving processes contribute significantly to environmental impact, particularly for yarns thinner than 100 dtex, whereas knitting tends to be more environmentally sustainable than weaving. Additionally, they emphasize that LCA results can vary considerably depending on the inclusion of dyeing, finishing, use phase, and end-of-life scenarios, further highlighting the complexity and case-specific nature of environmental assessments in the textile sector. In another study, Amicarelli et al. aimed to assess the environmental impacts associated with textile production processes and explored strategies for shifting from the traditional linear “take-make-waste” model to a more sustainable circular economy approach [59]. The authors note that current literature faces a significant limitation due to the limited number of studies and inconsistent methodologies, especially regarding the selection of impact categories, which complicates cross-study comparisons. Assessment of water, soil, and air use indicates that natural fibers, such as cotton, tend to have notable environmental impacts. This is primarily attributable to the extensive water consumption and considerable use of agricultural inputs associated with their production [60]. In contrast, synthetic fibers have a lower impact in terms of water and soil use but are more significant in terms of fossil fuel consumption. As expected, dyeing and finishing processes were found to be major contributors to environmental burdens due to their high energy demand and chemical use. Importantly, the study emphasizes that recycling and reuse strategies offer substantial environmental benefits, significantly reducing impacts when compared to landfilling or incineration scenarios.

In the end, LCA in the textile sector states that achieving a transition to a circular model in the textile industry requires coordinated actions across several areas. This includes encouraging the development and manufacture of materials designed for easy disassembly to support recycling. It also involves incorporating eco-design principles that consider future reuse or repair. In addition, initiatives such as second-hand markets and clothing exchange programs can extend the lifespan of garments and reduce the volume of textile waste sent to landfills.

While LCA provides a robust framework for identifying and quantifying the environmental impacts of textile products across their life cycle, sustainability assessments remain incomplete without considering economic performance. In this context, LCC extends the textile life cycle perspective to the economic dimension, enabling the evaluation of costs associated with textile products and processes throughout their entire lifespan.

In the textile sector, LCC typically considers a set of cost categories that reflect the economic implications of production and supply chain activities across the entire life cycle. These commonly include capital investment costs, such as machinery for spinning, weaving, knitting, dyeing, and finishing processes; operational costs, encompassing raw materials (e.g., natural and synthetic fibers), energy, water, chemicals, and labor; as well as maintenance and repair costs required to ensure continuous and efficient operation of production systems [61]. In this context, Mousavi et al. applied LCC to the textile sector by developing a simulation-based framework to assess economic performance across the full life cycle of textile products [62]. Their approach explicitly defined key LCC cost categories, including capital investment, operational, maintenance, and end-of-life costs, while relying on assumptions related to discount rates, inflation, and temporal variability. The study illustrates how LCC outcomes are highly sensitive to modeling choices, data uncertainty, and cost allocation procedures [62]. Other important factors taken into consideration during the LCC framework development are the end-of-life costs related to waste management, recycling, or disposal of textile products and production residues. The relative importance of these categories can vary substantially depending on the type of textile product, production technology, and geographical context. For example, Anceschi and colleagues explored the use of LCC to evaluate the sustainability of upcycling PET-polyurethane fabrics [63]. Through a relatively simple pyrolysis process, they successfully produced carbonaceous materials at a cost of approximately €1.73, which aligns with figures reported in the literature. Their findings demonstrate that upcycling can be a viable and cost-effective strategy for the valorization of difficult-to-treat textile waste, particularly synthetic composites. Similarly, Fidan et al. investigated the economic sustainability of producing denim fabric using fibers made from recycled PET bottles [64]. Their analysis compared recycled PET fibers with conventional PET fibers, evaluating variations in cost and impact depending on the proportion of recycled PET blended with cotton. The LCC results demonstrated that denim incorporating recycled PET fibers incurs higher production costs, primarily attributable to the additional processing required for recycled PET. As a result, it is more expensive than denim made with virgin PET. However, despite the higher costs, the study concludes that recycled PET offers a more sustainable alternative to both virgin PET and cotton in several environmental impact categories, suggesting strong potential for its use in more circular textile applications. Fatima et al. evaluated the environmental and economic sustainability of producing home textiles using r-PET fiber derived from post-consumer PET bottles [65]. The methodology covers the pre-fiber production stages, such as bottle collection, shredding, and pelletizing, and takes into account the environmental impacts related to material transportation. The PET flakes are then sent to an extrusion facility, where they are processed into r-PET fiber for use in home textile manufacturing. The study includes all stages from fiber production to finished fabric and reports a total production cost of USD 28,000 per ton. The LCC analysis reveals that 39.5% of this cost is attributable to dyeing and finishing processes.

**Figure 5:**
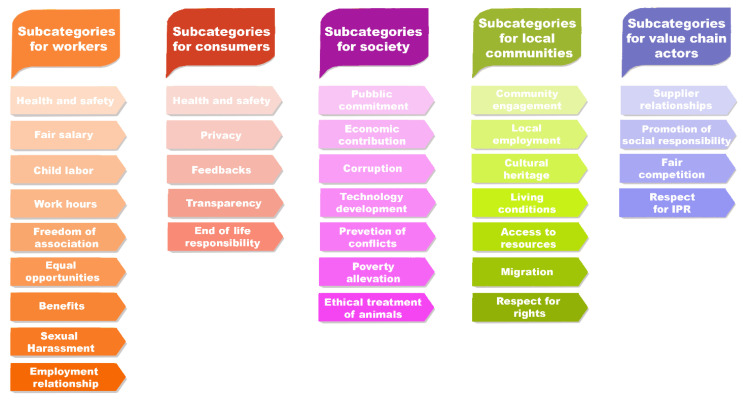
Frequency of use of S-LCA subcategories for textile [67].

Sustainability within the textile sector should include not only environmental and economic factors but also incorporate the social dimension. The textile value chain is intrinsically connected to various social concerns, such as labor conditions, workers’ rights, gender equity, and community well-being. These issues are especially pertinent in developing countries, where a significant portion of textile production occurs. To achieve this, the most explored methodology is the S-LCA. In a study by Fidan and co-workers, the S-LCA was applied in order to comprehensively examine critical social subcategories used in S-LCA to highlight the social sustainability of the textile sector [32]. Typically, workers represent the most frequently examined stakeholder category within S-LCA studies, whereas consumers receive the least attention. As previously discussed, in addition to stakeholder categories, sub-categories are also used for assessing the LCA. Sub-categories in S-LCA facilitate the evaluation of social impacts across various stakeholder groups. Examples include fair wages, occupational health and safety, working hours, and social security for workers, as well as access to information and product quality for consumers. Each sub-category enables a focused assessment of the social performance of a product system in relation to specific stakeholder needs and rights. The extent to which these sub-categories are addressed in the literature varies among different groups. Figure 5 illustrates the most commonly examined sub-categories within recent S-LCA studies in the textile sector.

In textile-related S-LCA case studies, stakeholder categories are typically operationalized through a structured selection of subcategories and context-specific indicators aligned with UNEP/SETAC guidelines. For example, within the “Workers” category, commonly applied indicators include fair wages (often assessed against living wage benchmarks or minimum wage compliance), occupational health and safety (evaluated through accident rates, exposure to hazardous substances, or safety certification schemes), working hours, social security coverage, and the presence of child or forced labor risks. These indicators are operationalized using a combination of site-specific primary data, supplier audits, sectoral reports, and risk-based databases such as the Social Hotspots Database (SHDB). At the same time, operationalization varies significantly depending on geographic location, production stage, and data availability. In the globally fragmented textile supply chain, particularly in polymer-based textile production, where upstream stages may involve petrochemical industries and downstream stages garment assembly in emerging economies, social risks differ considerably across regions. As a result, S-LCA applications often rely on proxy indicators or country-level risk data when company-specific information is unavailable. This contextual variability affects the robustness and comparability of results, highlighting that stakeholder categories in S-LCA should not be interpreted as fixed analytical units but as flexible frameworks requiring adaptation to specific supply chain configurations.

Among all the sub-categories studied, health and safety receive the greatest attention, followed closely by fair salaries. Other frequently assessed sub-categories include child labor, equal opportunities and non-discrimination, and working hours. Of course, these social themes are often examined in conjunction to provide a more comprehensive understanding of labor conditions and social risks throughout the product life cycle [66,67]. In a study by Lenzo and colleagues, the first application of the S-LCA to a textile product made in Sicily (Italy) was explored [68]. The study examined the production of wool and cashmere garments using a cradle-to-gate approach. The stakeholder groups considered were primarily workers and local communities, as they are seen as key representatives of the company’s social value within the territory. However, the analysis revealed that the company does not implement specific measures to prevent or mitigate social issues, nor does it promote proactive actions to safeguard its suppliers or other business partners. Implementing S-LCA would benefit the company by identifying suppliers and customers with strong social performance, supporting informed decisions, and promoting sustainability throughout the product life cycle. A recent study used the Sustainable Development Goals (SDGs) to assess social sustainability in the textile sector [69]. The study assessed the effects of textile products on SDGs using a T-shirt supply chain spanning five countries from China to the Netherlands. The analysis identified social risks related to health and well-being, affordable and clean energy, fair work and responsible production, especially in Bangladesh and Malaysia. A site-specific assessment found poor social performance at the spinning stage and other negative effects such as excessive working hours, unsafe living conditions and limited access to resources across all suppliers. In another interesting work, Muñoz-Torres and colleagues examined the key social impacts associated with the textile industry and assessed how effectively textile companies address these issues to enhance social sustainability within global supply chains [70]. The study conducted a comprehensive analysis by aligning the social indicators disclosed by textile companies with the social impact categories established by UNEP/SETAC. This evaluation was further supported by a comparison with data from the Social Hotspots Database (SHDB), focusing on both the upstream (input materials) and downstream (post-production) social impacts associated with textile products. The assessment found several social risks in different categories outlined by the S-LCA framework, with specific attention given to those connected to input materials. Importantly, the study expanded the stakeholder analysis beyond commonly examined groups such as workers and consumers, also including less frequently addressed stakeholders like local communities and vulnerable populations. The results revealed significant inconsistencies in the social information reported by companies across different life cycle stages. These discrepancies suggest that companies operating within complex and globalized supply chains often lack comprehensive visibility over social impacts. Accordingly, the authors promote conducting comprehensive S-LCA studies that encompass all stakeholders across the textile value chain, thereby enabling a more accurate and inclusive evaluation of social sustainability.

## 4. To What Extent Do Existing Studies Allow for Comparability and Integration Across Environmental, Economic, and Social Dimensions?

Despite the growing number of sustainability assessments applied to the textile sector, the extent to which existing studies allow for comparability and integration across environmental, economic, and social dimensions remains limited. While LCA, LCC and S-LCA are conceptually designed to address complementary pillars of sustainability, their application in the textile context is often fragmented, with methodological inconsistencies that hinder cross-study comparison and holistic interpretation.

One of the main barriers to comparability lies in the heterogeneous definition of system boundaries and functional units [71]. For instance, some studies include both fiber production and raw material acquisition within the same system boundary, which may lead to overlapping or ambiguous results [59,60]. Moreover, in textile sustainability studies, environmental assessments frequently adopt cradle-to-grave or cradle-to-gate approaches, whereas LCC and S-LCA applications often focus on selected life cycle stages or specific supply chain actors, depending on data availability and study objectives. For example, certain studies consider yarn or fabric production as the system boundary, whereas others define the boundary to encompass garment manufacturing or final product assembly [35,72,73,74]. A few studies even go beyond production to include transportation and retail stages [36,75,76]. In another case, Wang et al. included the use phase in their analysis, recognizing its significant contribution to overall environmental sustainability [77]. The results indicate that careful definition of system boundaries is important in textile studies. Variations in how these boundaries are set can influence the study outcomes and their interpretation. This misalignment complicates the direct comparison of results across sustainability dimensions and limits the possibility of integrated assessments. Differences in end-of-life modeling, allocation procedures, and treatment of recycling further exacerbate these inconsistencies, particularly in studies addressing polymer-based textiles and circular economy strategies.

Integration across dimensions is also constrained by differences in data types, scales, and methodological maturity. LCA typically relies on quantitative, process-based inventory data and standardized impact assessment methods, whereas LCC combines context-specific economic data that are sensitive to market conditions, discount rates, and temporal assumptions [78]. In contrast, S-LCA often employs qualitative or semi-quantitative indicators and risk-based databases to identify social hotspots at regional or sectoral levels [71]. These differences in spatial, temporal, and organizational scales make it challenging to aggregate or directly compare results across environmental, economic, and social domains, especially in globally fragmented textile supply chains.

Moreover, existing studies frequently present results from LCA, LCC, and S-LCA in parallel rather than within a unified analytical framework. While this side-by-side approach can offer complementary insights, it often lacks explicit mechanisms for evaluating trade-offs and synergies between sustainability dimensions. As a result, improvements in environmental performance may be associated with increased costs or unintended social impacts that are not systematically addressed within integrated decision-making frameworks. This limitation is particularly relevant in the context of circular economy strategies, where environmental benefits related to material recycling or resource efficiency may coincide with economic or social trade-offs along the supply chain.

Overall, current textile sustainability studies provide only partial comparability and predominantly qualitative integration across environmental, economic, and social dimensions. Advancing integrated sustainability assessment in the textile sector requires greater methodological alignment across LCA, LCC, and S-LCA, clearer reporting of assumptions and system boundaries, and the development of harmonized frameworks capable of capturing trade-offs across sustainability pillars. Such improvements are essential to enhance the robustness, comparability, and decision-making relevance of life cycle-based sustainability assessments in support of sustainable and circular transitions in the textile industry.

**Figure 6:**
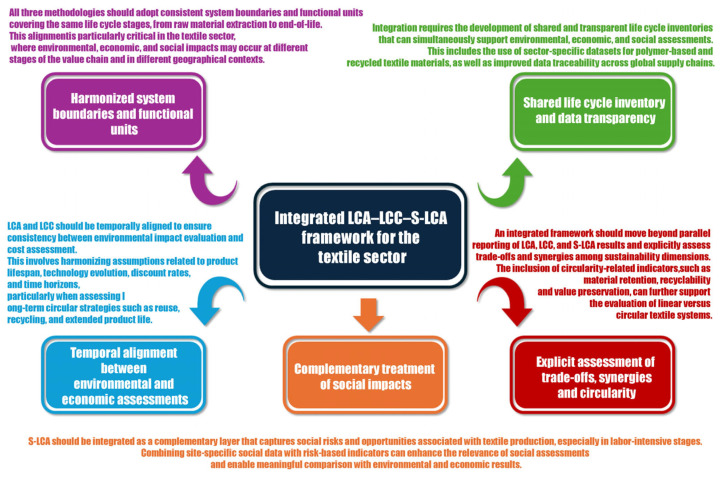
Conceptual overview of the key requirements for an integrated LCA–LCC–S-LCA framework applied to the textile sector.

A recap of the key requirements for an integrated LCA–LCC–S-LCA framework for the textile sector is presented in Figure 6.

Regarding the possible integration, despite the conceptual alignment of LCA, LCC, and S-LCA under the umbrella of life cycle thinking, their practical integration in textile sustainability assessment faces substantial operational barriers. One of the primary challenges concerns data compatibilities. Environmental LCAs typically rely on process-based, quantitative inventory data expressed in physical units (e.g., kg CO_2_ eq, MJ, m^3^ of water), whereas LCC is grounded in monetary flows influenced by company-specific accounting practices and market volatility. In contrast, S-LCA frequently uses qualitative or semi-quantitative indicators derived from country-level risk databases or stakeholder surveys. The heterogeneity of data types complicates aggregation and cross-dimensional comparison.

Indicator heterogeneity further limits integration. Environmental indicators are generally standardized and supported by internationally recognized impact assessment methods, while economic indicators depend on context-sensitive cost structures and social indicators often lack harmonized measurement frameworks. This asymmetry reduces methodological coherence across dimensions. Scale mismatches also represent a critical barrier. LCA is often conducted at the product or process level, LCC at the company or investment level, and S-LCA at regional or sectoral scales. In polymer-based textile supply chains, which are typically geographically fragmented across petrochemical production, fiber spinning, garment assembly, and distribution, aligning these scales becomes particularly complex. Finally, temporal inconsistencies undermine integrated assessment. LCA commonly applies static modeling assumptions for product lifetimes and recycling scenarios, whereas LCC depends on dynamic parameters such as discount rates, inflation, and future technology evolution. These divergent temporal logics make it difficult to coherently interpret environmental and economic performance within circular textile systems.

Addressing these practical barriers requires not only conceptual integration but also methodological harmonization, transparent reporting standards, and the development of hybrid assessment frameworks capable of managing multi-scale and multi-dimensional data structures.

## 5. What Limitations and Research Gaps Hinder the Effective Use of Life Cycle-Based Tools in Supporting Circular Strategies for Polymer-Based Textiles?

LCA, LCC and S-LCA are powerful tools designed to provide a comprehensive evaluation of environmental, economic, and social sustainability, respectively. When implemented accurately, these methodologies provide a comprehensive understanding of a product’s impact throughout its entire life cycle, taking into consideration all steps, from raw material extraction to end-of-life. Nonetheless, their practical application frequently encounters various challenges. To better understand the distinct roles and challenges of each life cycle-based sustainability assessment tool, Table 2 provides a comparative overview of LCA, LCC, and S-LCA. The table summarizes their primary goals, units of analysis, typical indicators, and methodological limitations, particularly within the context of the textile sector. This synthesis highlights the complementary nature of these methodologies and underscores the need for their integration in sustainability assessment frameworks.

For example, one of the principal strengths of LCA lies in its ability to identify environmental hotspots and quantify impacts such as greenhouse gas emissions, water consumption, and resource depletion. Nevertheless, the reliability of LCA results depends heavily on the quality and broadness of data. Many textile LCAs are affected by inconsistent data collection, unclear system boundaries, different functional units and production stages considered, and a lack of sector-specific databases. All these lead to difficult comparability between studies.

Similarly, LCC provides valuable insight into the total cost associated with a product throughout its life cycle. This is particularly important for assessing the economic viability of sustainable alternatives, in particular in recycling and recycled material productions. However, LCC is sensitive to market conditions, future cost assumptions, and uncertainties in estimating intangible costs and benefits, such as reputation or environmental burden. These limitations can make LCC results less robust or applicable across different economic contexts. Another critical challenge is the misalignment of temporal perspectives between LCA and LCC. While LCA typically evaluates environmental impacts over a static or standardized life cycle, LCC is highly sensitive to time-dependent variables such as discount rates, inflation, market dynamics, and technology evolution [26,28]. This temporal mismatch complicates the integrated interpretation of environmental and economic results and limits the robustness of combined sustainability assessments, especially when assessing long-term circular strategies for polymer-based textiles.

Despite being conceptually similar to other tools, S-LCA is the most complex and underdeveloped methodology. It helps assess rights, labor conditions, and community impacts to improve corporate social responsibility. However, methodological uncertainties persist due to qualitative, fragmented, and regionally gathered data. Many companies lack transparency and report unreliable social data, reducing the credibility and effectiveness of S-LCA studies. To further complicate the sustainability assessment, while each tool focuses on a specific pillar of sustainability, they are not always applied synergistically. This can result in conflicting outcomes; for instance, an economically viable product may have high environmental or social costs. Therefore, to pursue sustainable studies, it is essential to align LCA, LCC and S-LCA, along with other approaches not explicitly addressed in this review, under a unified framework. This framework will aim to systematically assess trade-offs and synergies across environmental, economic, and social dimensions. The need to align environmental, economic, and social assessments under a unified framework is crucial to support truly sustainable decision-making.

## 6. Conclusions

This review critically examined the application of life cycle-based sustainability assessment tools within the textile sector, with particular emphasis on polymer-based materials and circular economy strategies. The analysis confirms that these methodologies provide valuable and complementary insights into the environmental, economic, and social dimensions of textile sustainability. However, their potential to support robust and integrated decision-making remains only partially realized due to methodological fragmentation and data-related limitations. LCA represents the most mature and widely adopted tool in textile sustainability assessments, particularly for identifying environmental hotspots associated with fiber production, energy use, and end-of-life scenarios. Nevertheless, significant variability in system boundaries, functional units, data sources, and end-of-life modeling continues to undermine result comparability, especially for polymer-based textiles where circularity assumptions play a decisive role in shaping environmental outcomes. On the contrary, LCC and S-LCA remain underutilized and methodologically heterogeneous, despite their high relevance for evaluating the economic feasibility and social implications of sustainable and circular textile systems. LCC applications are strongly influenced by context-specific assumptions and market dynamics, limiting cross-study comparability, while S-LCA is constrained by qualitative indicators, limited site-specific data, and the lack of harmonized impact pathways. As a result, economic and social dimensions are often addressed in isolation or as secondary analyses, rather than being fully integrated into sustainability decision-making. Finally, this review would like to emphasize that existing studies rarely achieve true integration across environmental, economic, and social dimensions. Although LCA, LCC, and S-LCA are conceptually aligned through life cycle thinking, they are most often applied in parallel rather than within a unified analytical framework. This limits the ability to systematically assess trade-offs and synergies, particularly in the context of circular economy strategies for polymer-based textiles, where environmental benefits may be accompanied by economic or social challenges along the value chain.

Overall, this review highlights the urgent need for greater methodological alignment, transparency, and standardization in life cycle-based sustainability assessments applied to the textile sector. Future research should prioritize the development of sector-specific databases for polymeric and recycled textile materials, the advancement of quantitative and site-specific S-LCA approaches, and the harmonization of temporal and system boundary assumptions across LCA and LCC. Moreover, integrating explicit circularity indicators within life cycle-based frameworks will be essential to consistently evaluate and compare linear and circular textile systems.

Looking forward, advancing sustainability assessment in the textile industry will require a shift from fragmented evaluations toward integrated, decision-oriented frameworks capable of capturing environmental, economic, and social trade-offs in a coherent manner. Such an evolution is crucial not only for improving the scientific robustness of sustainability assessments but also for enabling policymakers, industry stakeholders, and designers to actively support the transition toward truly sustainable and circular polymer-based textile systems.

## Figures and Tables

**Table 2:** Comparison of LCA, LCC, and S-LCA methodologies in the context of textile sustainability assessment.

Dimension	LCA	LCC	S-LCA
Primary Goal	Assess environmental impacts across the product’s entire life cycle.	Quantify total costs of ownership over a product’s lifespan.	Analyze social and socio-economic impacts across all life cycle stages.
Focus Area	Environmental sustainability	Economic sustainability	Social sustainability
Key indicators	GHG emissions (CO_2_ eq), energy & water use, eutrophication, toxicity	Initial investment, operating & maintenance costs, disposal costs	Fair wages, occupational safety, child labor, access to services
Standards	ISO 14040/14044, EU Product Environmental Footprint (PEF)	ISO 15686-5 (adapted from construction industry)	UNEP/SETAC Guidelines (2009, 2020)
Strengths	Scientifically robust; widely adopted in environmental policy	Reveals medium- and long-term costs; supports sustainable procurement	Emphasizes human rights, community impacts, and ethical sourcing
Challenges	Inconsistent system boundaries, poor or hidden data, complex impact modeling	Unpredictable input data, assumption-heavy, lacks treatment of externalities	Lack of suitable data, qualitative indicators and harmonized databases
Textile Applications	Impact assessment of materials/processes/end-of-life (e.g., cotton vs. PET)	Cost–benefit analysis of sustainable textiles (e.g., rPET denim)	Evaluation of labor conditions along the supply chain

## Data Availability

No new data were created or analyzed in this study. Data sharing is not applicable to this article.
